# Integrating network pharmacology, molecular docking and experimental verification to explore the therapeutic effect of piceatannol on rheumatoid arthritis

**DOI:** 10.3389/fimmu.2026.1804844

**Published:** 2026-05-25

**Authors:** Mingyi Yang, Honghao Ren, Peng Xu, Xiaodong Ren, Yirixiati Aihaiti, Pengfei Wen, Lin Liu, Ke Xu, Ming Zhang, Weikun Hou, Zhi Yang, Yani Su

**Affiliations:** 1 Department of Joint Surgery, Honghui Hospital, Xi’an Jiaotong University, Xi’an, Shaanxi, China; 2 Xi’an Key Laboratory of Pathogenesis and Precision Treatment of Arthritis, Xi’an, Shaanxi, China; 3 Department of General Practice, Honghui Hospital, Xi’an Jiaotong University, Xi’an, Shaanxi, China; 4 Department of Radiotherapy, Tangdu Hospital, The Fourth Military Medical University, Xi’an, Shaanxi, China

**Keywords:** AIA, GEO, network pharmacology, piceatannol, rheumatoid arthritis

## Abstract

**Objective:**

Piceatannol (PIC) exhibits antioxidant and anti-inflammatory activities. This study integrates network pharmacology and experimental validation to investigate its potential role in rheumatoid arthritis (RA).

**Methods:**

PIC targets were predicted using public databases. RA-related differentially expressed genes (DEGs) were identified from Gene Expression Omnibus (GEO) datasets (|logFC| ≥ 1 and P-value < 0.05). Intersection genes were analyzed via protein-protein interaction (PPI) network (hub gene selection), molecular docking (binding affinity < -5.0 kcal/mol as threshold), ConnectivityMap and molecular dynamics simulation. Experimental validation included CCK8, flow cytometry, real-time quantitative PCR (RT-qPCR), Western blotting, and an adjuvant-induced arthritis (AIA) rat model.

**Results:**

35 intersecting genes were identified, from which 6 hub genes (SYK, CXCL8, TNF, NFKB1, PPARG, and CASP8) were selected. PIC showed stable binding to all hub genes (affinities: -5.6 to -7.8 kcal/mol). ConnectivityMap suggested a regulatory relationship between PIC and SYK. Molecular dynamics simulations demonstrate that the PIC–SYK complex maintains stable structural integrity. Experimental validation showed that PIC reduced MH7A cell viability, induced G2/M arrest and apoptosis, and downregulated mRNA levels of SYK, NFKB1, and CASP8, consistent with predictions. *In vivo*, PIC alleviated AIA severity.

**Conclusion:**

These preliminary findings suggest that PIC exerts therapeutic effects in RA models, potentially via SYK/NFKB1/CASP8. The study provides a theoretical basis for further evaluation of PIC in RA, while acknowledging the exploratory nature of network pharmacology and preclinical models.

## 1 Introduction

Rheumatoid arthritis (RA) is a chronic systemic autoimmune disorder marked by symmetric polyarticular synovitis as its hallmark feature. The condition involves inflammatory cell infiltration that contributes to degenerative changes in the synovial membrane, articular cartilage, and underlying bone structures (1, 2). The synovium serves as the primary site of inflammation in RA. Persistent synovitis results in joint surface erosion, culminating in deformities and functional impairment, which ultimately contribute to chronic disability and reduced lifespan (3). Affected joint tissues undergo continuous erosion, compromising joint architecture and resulting in stiffness or, in severe cases, overt deformity (4). Notably, phenotypic alterations in RA fibroblast-like synoviocytes (RA-FLS) exhibit a positive correlation with disease chronicity. These changes promote macrophage infiltration into the synovium and exacerbate cartilage destruction (5). Targeting FLS and their key signaling pathways has therefore emerged as a promising therapeutic approach for RA (6). Elucidating the mechanisms driving FLS invasiveness may uncover novel treatment targets aimed at mitigating inflammation and preventing bone erosion in RA.

Piceatannol (PIC) is a polyphenolic stilbene compound that occurs in two primary forms: one of natural origin, present in various commonly consumed human foods, and the other as a metabolite derived from resveratrol (7). Due to its potent antioxidant, anti-inflammatory, PIC has recently been able to attract the attention of several scientists all over the world. The antioxidant activity of PIC is based on the presence of hydroxyl groups on its stilbene rings. It has a high capacity for scavenging lipid peroxyl radicals. The semiquinone radicals from PIC have been discovered to be more stable. PIC has a protective effect on the development of osteoarthritis. PIC inhibited the activation of the core NF-κB by activating the nuclear factor erythroid 2 related factor 2 (Nrf2) and enzyme heme oxygenase 1 (HO-1) pathway (8). PIC was found to rescue the uncoupling protein 1 mRNA expression induced by isoproterenol in 10T1/2 adipocytes, which was suppressed by LPS-activated macrophages, suggest that PIC may attenuate the pathologic inflammation triggered by adipose tissues (9). PIC exhibits a potential protective effect against Indo-induced gastric ulcers by the antioxidant, anti-inflammatory, and angiogenic mechanisms (10). However, despite these broad effects, the specific role of PIC in RA—particularly its impact on RA-FLS and the underlying molecular mechanisms—has not been fully explored. This knowledge gap hinders the rational development of PIC as a potential anti-RA agent.

**Figure 1:**
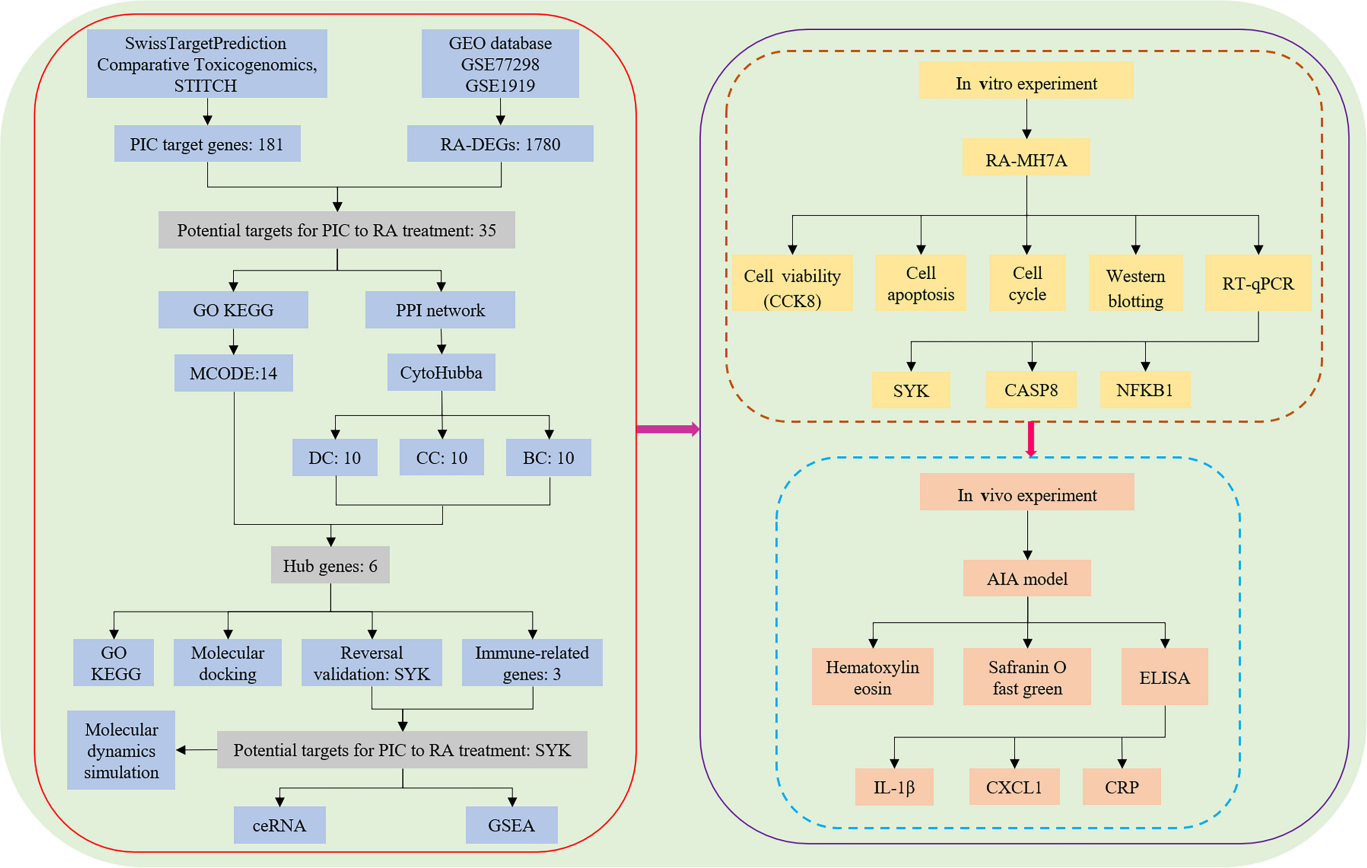
The flowchart of this study.

Given that RA is a complex, multi−gene disease and PIC likely acts on multiple targets, a comprehensive research approach is warranted. Bioinformatics has become indispensable in medical research (11). Network pharmacology—a subfield of bioinformatics—uses drug−target networks to systematically analyze pharmacological actions, making it well suited for predicting the molecular targets and pathways of natural compounds such as PIC in complex diseases like RA (12). Unlike traditional single−target approaches, network pharmacology can identify unexpected interactions and prioritize hub targets for experimental validation. Microarray technologies combined with bioinformatics have proven effective in discovering disease biomarkers (13). Accordingly, this study integrates network pharmacology with experimental validation (cell−based assays and an animal model of RA) to identify potential targets of PIC and verify the predicted effects on RA−FLS behavior and *in vivo* disease progression. This integrative framework bridges computational predictions with biological evidence, providing a robust basis for uncovering novel therapeutic mechanisms of PIC in RA. The research design and analytical workflow are summarized in **Figure 1**.

## 2 Materials and methods

### 2.1 Potential targets for PIC in the treatment of RA

Putative target genes of PIC were predicted using the SwissTargetPrediction database (14), the Comparative Toxicogenomics database (15), and the STITCH database (16–18). Gene expression profiles of RA synovial tissues and normal synovial tissues were obtained from the Gene Expression Omnibus (GEO) under accession numbers GSE77298 and GSE1919. These datasets were selected because they both contain transcriptomic data from human synovial tissues of RA patients and healthy controls, are derived from similar microarray platforms (Affymetrix Human Genome U133 Plus 2.0), and collectively provide a reasonable sample size (21 RA, 12 normal) to improve statistical power. The dataset GSE77298 comprises 16 RA and 7 normal synovial samples, while GSE1919 includes 5 RA and 5 normal synovial samples. To eliminate technical batch effects while preserving biological variability, raw expression data from the two datasets were integrated and normalized using the ComBat algorithm as implemented in the sva package in R. The batch variable was assigned according to dataset origin (GSE77298 as batch 1, GSE1919 as batch 2). The biological condition of interest (RA vs. normal) was treated as a covariate in the model matrix, ensuring that biological differences were retained during the batch effect correction process. Differential gene expression analysis was conducted using the “limma” package in R to identify differentially expressed genes (DEGs), with screening criteria set as |logFC| ≥ 1 and P-value < 0.05 (18, 19). Potential therapeutic targets of PIC for RA treatment were identified by taking the intersection between PIC target genes and DEGs (17).

### 2.2 GO and KEGG enrichment analysis

Gene ontology (GO) and Kyoto encyclopedia of genes and genomes (KEGG) enrichment analysis were performed on the overlapping genes between PIC target genes and DEGs using the DAVID database, with a significance threshold set at P-value < 0.05. The GO analysis encompassed three functional categories: biological process, cellular component, and molecular function.

### 2.3 Construction and analysis of PPI network

PPI analysis of the overlapping genes between PIC target genes and DEGs was conducted using the STRING database (20), with interactions meeting a minimum confidence score of ≥ 0.4 considered statistically significant. The resulting protein-protein interaction (PPI) network was visualized using Cytoscape (version 3.8.0). Module analysis was carried out using the MCODE plugin within Cytoscape, and the two highest−ranked module genes were selected for subsequent analysis. Central genes were then identified via the CytoHubba plugin based on three topological metrics—degree centrality (DC), closeness centrality (CC), and betweenness centrality (BC)—with the top ten genes from the PPI network ranked by each measure being considered as central genes. The hub genes were subsequently defined as the intersection of the aforementioned central genes and the top two module genes. Functional enrichment analysis of these key genes was performed using the DAVID database, encompassing GO terms and KEGG pathways.

### 2.4 Molecular docking of the PIC and hub genes

The three-dimensional structure of PIC was generated from its two-dimensional representation using ChemOffice software, yielding a small-molecule ligand suitable for docking studies. Protein identifiers corresponding to the hub genes were retrieved from the UniProt database. The three-dimensional structures of these proteins were subsequently acquired from the Protein Data Bank (PDB) based on their respective IDs. Using PyMOL software, non-relevant small molecules and water molecules were removed from each protein structure to prepare clean receptor models. Prior to docking, each protein structure was preprocessed using AutoDockTools (ADT) to add polar hydrogen atoms, assign Gasteiger charges, and merge non-polar hydrogens. Energy minimization was performed using the conjugate gradient algorithm with a maximum of 500 steps and a root-mean-square gradient tolerance of 0.01 kcal/(mol·Å) to relieve steric clashes and optimize the geometry of both the ligand and receptor. Both the PIC ligand and the processed protein receptors were converted into PDBQT format using AutoDockTools, which was also used to define the active binding pockets of the receptors. For each docking simulation, a grid box was centered on the predicted or crystallographically defined binding site of the target protein. The exhaustiveness parameter was set to 16 for all docking runs. Molecular docking was performed using Vina software, which employs a hybrid scoring function combining empirical and knowledge-based terms to estimate binding free energies. The binding affinity (ΔG) was calculated in kcal/mol, and a threshold of < -5.0 kcal/mol was used to define a significant interaction, based on commonly accepted criteria for ligand-protein docking. Docking was performed to simulate binding interactions between the PIC ligand and each protein receptor, generating corresponding docking output files. Finally, the docking results were visualized using PyMOL software.

### 2.5 Signature reversal analysis of PIC as a potential therapeutic agent for RA

The ConnectivityMap database was employed to predict targeted therapeutic agents corresponding to the identified hub genes. This approach was further utilized to investigate whether the hub gene could be inversely associated with PIC, thereby assessing the potential of PIC to serve as a therapeutic agent for RA through signature reversal-based drug repositioning.

### 2.6 Molecular dynamics simulation

Molecular dynamics simulations were performed using GROMACS 2023.2 software to evaluate the binding stability and binding degree of the ligand with the target protein. The protein force field was set to amber99sb-ildb, and the ligand force field was set to gaff. TIP3P water molecules under the SPCE model were used as the solvent, and a cubic water box of 6×6×6 nm was constructed; ions were added to neutralize the net charge of the system. Energy minimization was carried out in two steps: first, using the steepest descent method for up to 10, 000 iterations, followed by the conjugate gradient method for an additional 10, 000 iterations, with a convergence threshold of energy change less than 1000 kJ/mol/nm. Subsequently, NVT and NPT ensemble equilibration were each performed for 1, 000, 000 steps with a step size of 2 fs. After equilibration, a 100 ns molecular dynamics simulation was conducted for the PIC–hub gene complex, and the trajectories were analyzed for root-mean-square deviation (RMSD), root-mean-square fluctuation (RMSF), radius of gyration (Rg), solvent-accessible surface area (SASA), number of protein–ligand hydrogen bonds, and free energy landscape. A flatter RMSD curve indicates greater stability of the complex; smaller RMSF values reflect lower residue flexibility and more stable structure; a small Rg value indicates that the system remains compact during the simulation; changes in SASA reflect protein folding status; and a higher number of hydrogen bonds generally implies stronger binding. The Gibbs free energy was calculated using the g_sham.py and xpm2txt.py scripts, and free energy landscapes were plotted based on RMSD, Rg, and free energy values.

### 2.7 Identification of immune−related genes and PIC target genes

The GeneCards database consolidates comprehensive information on all currently annotated and predicted human genes, integrating genomic, transcriptomic, and proteomic data from approximately 125 network sources. Given that RA is an autoimmune disorder, we conducted a targeted screen within GeneCards to identify potential protein-level therapeutic targets of PIC for RA, with emphasis on immune−related genes. The intersection between the immune−related genes with an immune score > 60 and the previously identified hub genes was determined. This was ultimately used to identify the most promising candidate targets for PIC in the treatment of RA.

### 2.8 Construction of the ceRNA network and screening of key regulatory axes

To construct a competitive endogenous RNA (ceRNA) network for target genes that can predict PIC in reverse, miRNA targeting predictions were first performed using the TargetScan, miRWalk, and miRDB databases. To improve prediction reliability, only the overlapping results from all three databases were retained. The identified miRNAs were then used to predict target lncRNAs through the starBase and LncBase databases (21). The lncRNA expression dataset GSE103578, which includes three RA and three normal synovial samples, was downloaded from the GEO database. Differential expression analysis was carried out using the “limma” package in R to identify both DEGs and differentially expressed lncRNAs (DELncs), with the screening thresholds set at |logFC| ≥ 1 and P−value < 0.05. Potential therapeutic lncRNAs for PIC in RA treatment were identified by intersecting the lncRNAs predicted via miRNA targeting with the DELncs obtained from GSE103578. Finally, a comprehensive ceRNA network was constructed, incorporating these candidate lncRNAs along with their corresponding miRNAs and mRNAs.

### 2.9 Gene set enrichment analysis

Gene set enrichment analysis (GSEA) was performed on the genes obtained from the intersection of immune−related genes and hub genes, and the intersection gene could also be inversely predicted as targets of PIC. A P-value<0.05 was applied to determine statistical significance.

### 2.10 Cell culture

Human FLS of MH7A were purchased from the Shanghai Cell Bank of the Chinese Academy of Sciences. The cells were cultured in DMEM medium, supplemented with 10% fetal bovine serum and 1% penicillin/streptomycin. The cells were incubated in a 37 °C, 5% CO2 incubator. The MH7A cells were divided into six groups: control, LPS, PIC5μM, PIC10μM, PIC20μM and PIC40μM.

### 2.11 Cell viability assay

Cell viability was determined using Cell Counting Kit-8 (CCK-8, TargetMol, China). Cells were seeded at a density of 2000 cells per well in a 96-well plate. After overnight incubation, MH7A cells were treated with different concentrations of PIC (0, 5, 10, 20, and 40 μM) for 24 to 96 hours. 10 μL of CCK-8 solution was added to each well, and incubated for 1 hour. The optical density (OD) was measured at a wavelength of 450 nm.

### 2.12 Cell apoptosis and cell cycle measurement

After treating MH7A cells with different concentrations of PIC (0, 5, 10, 20 and 40 μM) and LPS at 1 μg/mL for 24 hours, the cells were enzymatically digested, washed with PBS, and according to the instructions of the apoptosis detection kit (KeyGEN BioTECH, China, No. KGA108), flow cytometry was performed using Annexin V-FITC/PI. And the cell cycle detection kit (Soralbio, China, No. CA1510), the proportion of cell apoptosis and cell cycle was detected using a flow cytometer. Flow cytometry was performed on a CytoFLEX flow cytometer equipped with 488 nm and 638 nm lasers. For each sample, 10, 000 events were acquired.

### 2.13 Western blotting

Extract total protein using RIPA lysis buffer, quantify the protein, and perform standard Western blotting. Evaluate the protein levels using appropriately diluted specific primary and secondary antibodies: BCL-2 (Bioss, No. bs-20351R), BAX (Bioss, No. bs-0127R), ACTB (Proteintech, No. 20536-1-AP). The dilution ratio of BCL-2 and BAX is 1:500, and the dilution ratio of ACTB is 1:1000. Display the signals using ECL detection reagent (Millipore, USA). All blots were standardized using the ACTB signal.

### 2.14 Real-time quantitative PCR

Total RNA was extracted from cells using TRIzol reagent (Life Technologies Invitrogen, USA) and then reverse transcribed into cDNA. According to the manufacturer’s instructions, standard Real-time quantitative PCR (RT-qPCR) was performed using ChamQ Universal SYBR qPCR Master Mix (Vazyme, China). The relative expression levels of all genes were normalized with β-actin as the reference, and the RT-qPCR results were evaluated using the 2^–ΔΔ^*^Ct^* method. The primers were synthesized and purchased by Shanghai Sangon Biotech (**Table 1**).

**Table 1:** Primer sequences for RT-qPCR.

Genes	Forward	Reverse
β-actin	TTAATAGTCATTCCAAATATGA	GGGACAAAAAAGGGGGAAGG
SYK	CATGGAAAAATCTCTCGGGAAGA	GTCGATGCGATAGTGCAGCA
CASP8	GGTGCTACCATCGTGAGAGT	GGTTCTTGCTTCCTTTGCGG
NFKB1	GATCCATATTTGGGAAGGCCTGA	CAGTGCCATCTGTGGTTGAAA

### 2.15 The construction of the AIA model

This study was conducted in accordance with the guidelines approved by the Animal Ethics Committee of Xi’an Jiaotong University (Approval No. XJTUAE2025-3861). Female Sprague-Dawley rats (6–8 weeks old), obtained from the Experimental Animal Center of Xi’an Jiaotong University, were acclimatized for one week prior to experimentation. Experimental arthritis—adjuvant-induced arthritis (AIA)—was induced by injecting 100 μL of complete Freund’s adjuvant (CFA; Sigma-Aldrich, USA) into the right hind paw under sodium pentobarbital anesthesia. On day 7, a secondary challenge was performed by administering another 100 μL of CFA into the same paw. The rats (n = 30) were randomly assigned to three groups: control, AIA and AIA + PIC, using a random number table generated by SPSS. Randomization and group allocation were performed by an investigator not involved in subsequent experiments. Starting on day 14, rats in the AIA + PIC group received daily intragastric administration of PIC (20 mg/kg/day) for 14 consecutive days (22, 23). The experimenter performing drug administration was blinded to group allocation. On day 28, all animals were anesthetized with sodium pentobarbital. Blood samples were collected via the abdominal aorta, stored overnight at 4 °C, and subsequently processed for serum isolation. Histopathological evaluation of synovial hyperplasia, immune cell infiltration, and cartilage damage was performed by two independent observers using a double-blind scoring system, with both observers unaware of group assignment. Throughout the experiment, animals were monitored daily for signs of pain, distress, or morbidity. All procedures were performed under anesthesia, and every effort was made to minimize suffering.

### 2.16 Hematoxylin-eosin staining, safranin O-fast green staining

Synovial and cartilage samples were collected from rats, fixed, embedded, and sectioned. Hematoxylin-eosin and safranin O-fast green staining were performed per manufacturer’s instructions. Images were captured using a Leica microscope. Histopathological evaluation was conducted by two independent blinded observers using a 0–4 scoring system for synovial hyperplasia (cell layers), inflammatory infiltration (density), and cartilage destruction (safranin O loss and erosion). The sum of the three subscores (maximum 12) was used for analysis.

### 2.17 Enzyme-linked immunosorbent assay

The levels of IL-1β, CXCL1 and CRP in the cell culture supernatant and rat serum were determined using Enzyme-linked immunosorbent assay (ELISA) kits (Boster, China). The concentrations of each cytokine in the wells were calculated based on the standard curve, and the actual concentrations were calculated according to the dilution factor.

### 2.18 Statistical analysis

Bioinformatics analysis was conducted using R 4.0.5, Cytoscape 3.8.0 and the online bioinformatics drawing tool Microbiome. The experimental data were plotted using Graphpad Prism 8 software, and statistical analysis was performed using SPSS 22.0. Normality of the data was assessed using the Shapiro–Wilk test, and homogeneity of variances was evaluated using Parametric test. The two groups were compared using the T-test. For comparisons involving more than two groups, one-way analysis of variance (ANOVA) was employed, followed by Tukey’s *post hoc* test for multiple pairwise comparisons when the overall ANOVA was significant. And all experiments were repeated at least three times. The data were presented as mean ± standard deviation. In the AIA model, there were 10 rats in each group, which fully met the statistical requirement of n ≥ 3. Difference analysis, enrichment analysis and experimental verification, a P-value < 0.05 was considered statistically significant. The difference analysis and enrichment analysis did not undergo multiple testing correction.

## 3 Results

### 3.1 Multi−level research strategy

To systematically investigate the therapeutic mechanism of PIC in RA, we adopted a multi−layer integrative strategy. First, we identified overlapping genes between PIC targets and RA−related DEGs to obtain candidate targets. Next, PPI network analysis and hub gene selection were performed to prioritize the most functionally central nodes among these candidates. Molecular docking was then used to validate the direct binding potential between PIC and each hub gene. Recognizing that RA is an immune−driven disease, we further intersected the hub genes with immune−specific genes to focus on immunologically relevant targets. Finally, to explore upstream regulatory mechanisms, we constructed a ceRNA network for the key immune−related hub gene SYK, thereby linking the predicted targets to non−coding RNA regulation. This stepwise approach ensures that each layer of analysis addresses a distinct but complementary question, and the results are integrated into a coherent mechanistic model.

### 3.2 Potential targets for PIC in the treatment of RA

**Figure 2:**
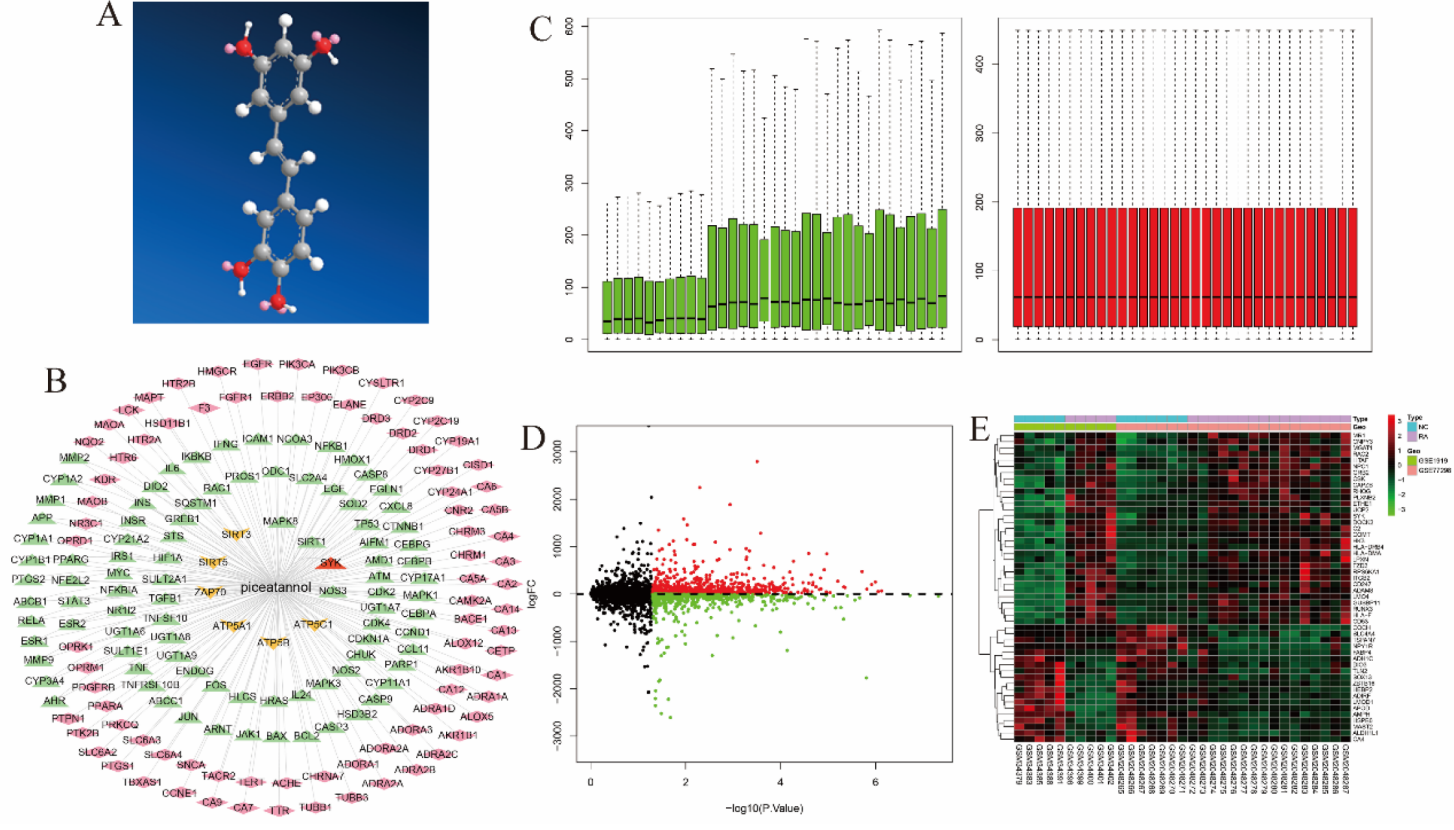
The target genes of PIC and DEGs of RA. **(A)** The chemical structure of PIC; **(B)** 181 unique PIC target genes; **(C)** Batch correction; **(D)** Volcano plot of DEGs, red indicates upregulation, and green indicates downregulation.; **(E)** Heatmap of DEGs, red indicates upregulation, and green indicates downregulation.

**Figure 3:**
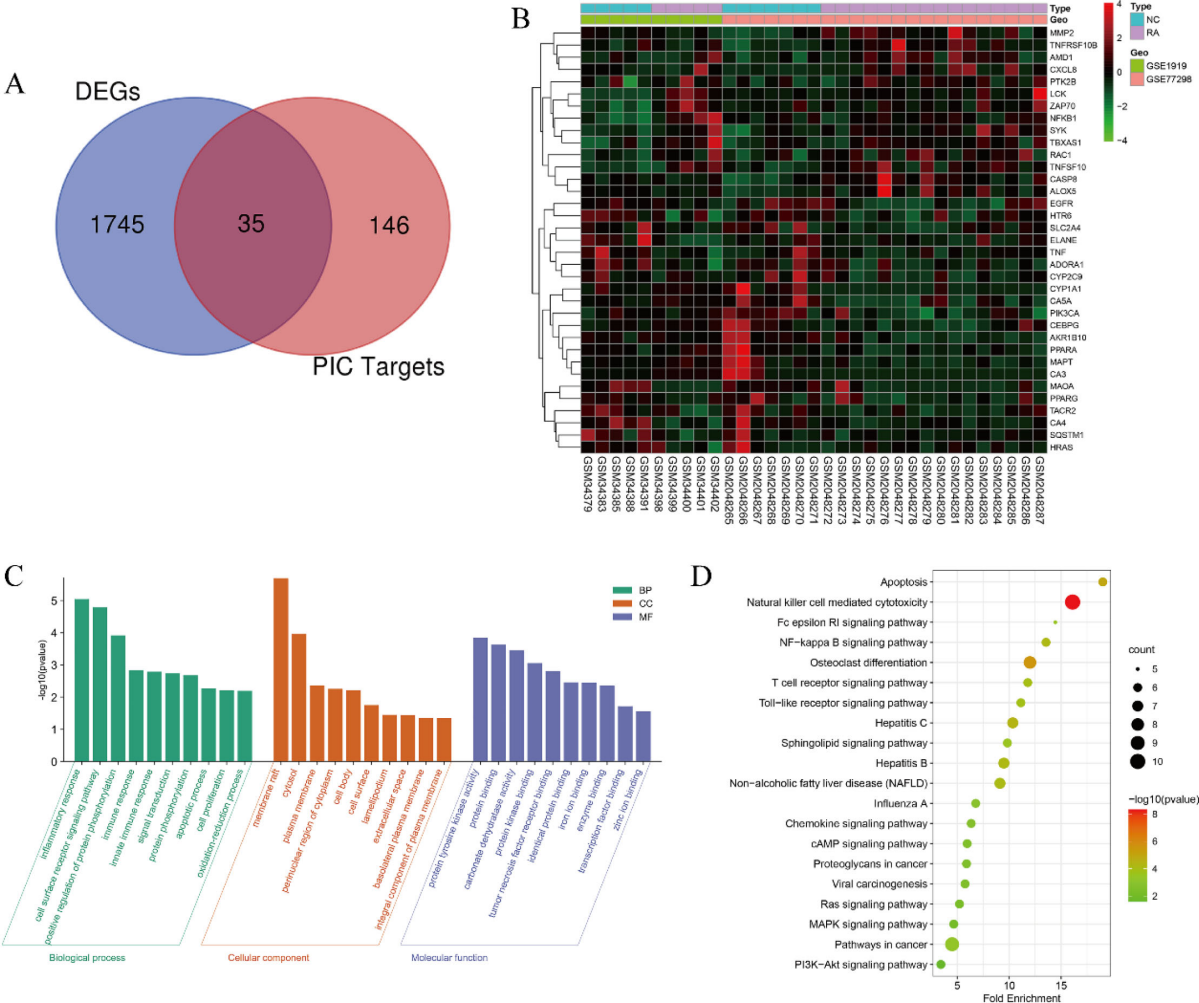
Potential therapeutic targets for PIC in the treatment of RA and enrichment analysis. **(A)** 35 potential therapeutic targets for PIC in the treatment of RA; **(B)** Heatmap of 35 candidate genes, red indicates upregulation, and green indicates downregulation; **(C)** GO enrichment analysis, include biological process, cellular component, and molecular function; **(D)** KEGG enrichment analysis.

The chemical structure of PIC was retrieved from the PubChem database (24) (**Figure 2A**). A total of 101, 94, and 10 target genes of PIC were predicted using the SwissTargetPrediction, Comparative Toxicogenomics, and STITCH databases, respectively. The predicted targets from all three databases showed mutual overlap, resulting in a combined set of 181 unique PIC target genes (**Figure 2B**). After integrating the two datasets, GSE77298 and GSE1919, batch correction was performed to reduce inter-batch differences (**Figure 2C**). A total of 1780 DEGs were identified. The volcano plot (**Figure 2D**) revealed a substantial number of upregulated (red) and downregulated (green) genes in RA synovial tissues compared with normal controls. The heatmap (**Figure 2E**) showed distinct clustering of RA samples versus normal controls, with clear separation of gene expression patterns, confirming that the identified DEGs effectively discriminate RA from healthy synovial tissue. Intersection of the PIC target genes with the DEGs yielded 35 overlapping genes, which were identified as potential therapeutic targets for PIC in the treatment of RA (**Figure 3A**). A heatmap depicting the expression profiles of these 35 candidate genes (**Figure 3B**) demonstrated that most of these genes were consistently upregulated or downregulated across RA samples relative to controls, suggesting coordinated expression changes that may be relevant to RA pathogenesis and amenable to modulation by PIC.

### 3.3 GO and KEGG enrichment analysis

Enrichment analysis of the 35 overlapping genes between PIC target genes and RA-related DEGs was performed using DAVID. Among the biological processes, several terms closely linked to RA pathogenesis were significantly enriched, including inflammatory response, immune response, innate immune response, apoptotic process, cell proliferation, and positive regulation of protein phosphorylation. These processes are known to drive synovial inflammation, FLS hyperplasia, and joint destruction in RA. For cellular components, the genes were predominantly localized to the plasma membrane, extracellular space, and cytoplasm, reflecting their roles in signal reception and transduction. Molecular function analysis showed protein tyrosine kinase activity, protein kinase binding, tumor necrosis factor receptor binding, and enzyme binding, suggesting that PIC may interfere with key kinase-driven signaling cascades in RA. KEGG pathway enrichment revealed that the candidate genes are primarily involved in several pathways with established relevance to RA pathology, including the NF-kappa B signaling pathway, apoptosis, T cell receptor signaling pathway, Toll-like receptor signaling pathway, osteoclast differentiation, natural killer cell-mediated cytotoxicity, and chemokine signaling pathway. Additionally, pathways related to broader inflammatory and oncogenic contexts—such as MAPK signaling, PI3K-Akt signaling, Ras signaling, and pathways in cancer—were also enriched, reflecting the multi-faceted nature of RA. Notably, the enrichment of NF-κB and apoptosis pathways aligns with PIC’s known anti-inflammatory and pro-apoptotic activities, suggesting that PIC may ameliorate RA by suppressing synovial inflammation and promoting FLS apoptosis. The presence of osteoclast differentiation further implies a potential role for PIC in mitigating RA-associated bone erosion. Overall, these enriched pathways provide a mechanistic basis for the therapeutic effects of PIC in RA (**Figures 3C, D**).

### 3.4 Construction and analysis of PPI network

**Figure 4:**
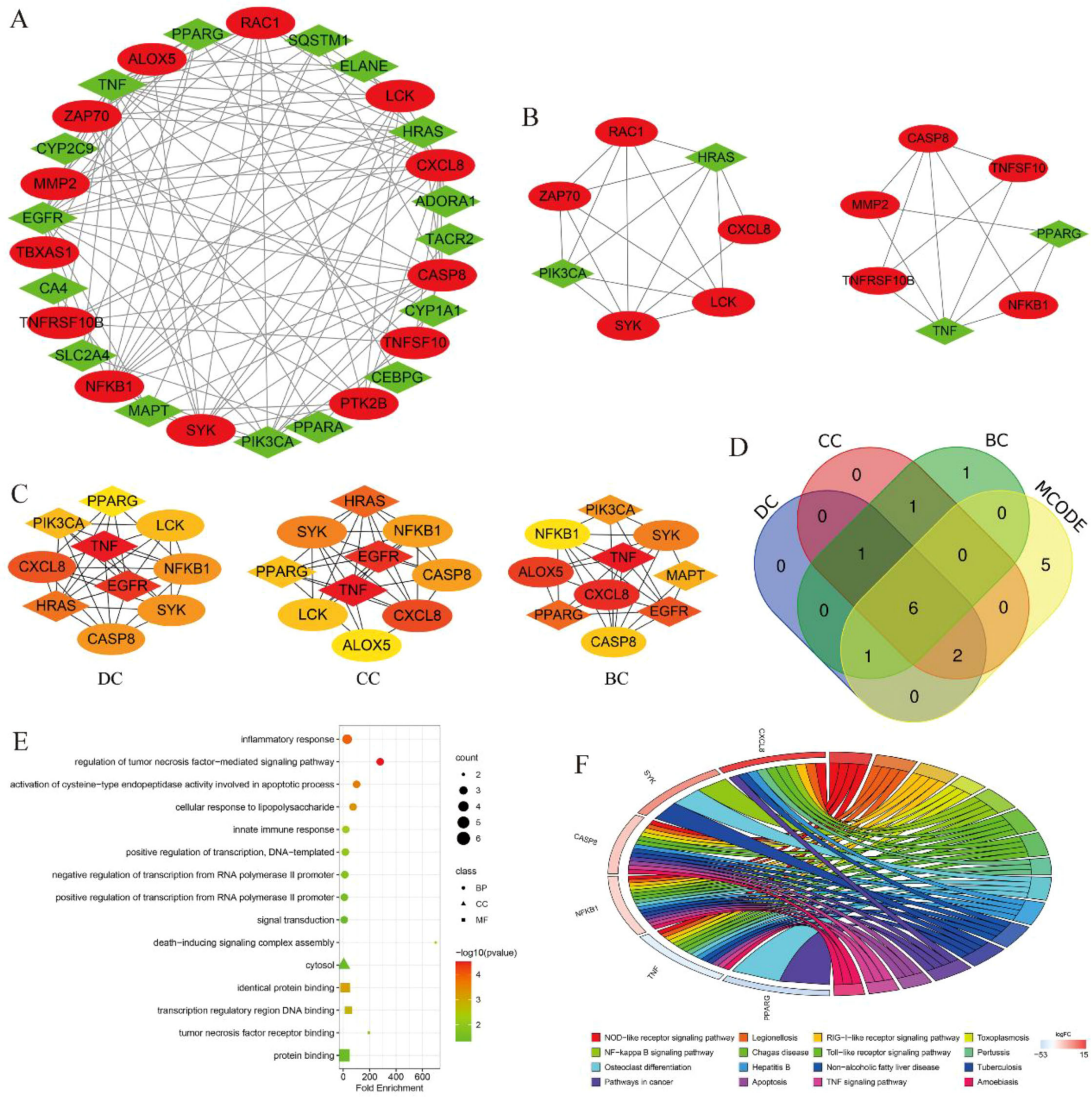
PPI network construction and analysis. **(A)** PPI network; **(B)** Module analysis, two highest−ranked module genes; **(C)** Central genes analysis, degree centrality, closeness centrality, and betweenness centrality, with the top ten genes from the PPI network; **(D)** Six hub genes, the intersection of the aforementioned central genes and the top two module genes; **(E)** GO enrichment analysis, include biological process, cellular component, and molecular function; **(F)** KEGG enrichment analysis.

Within the PPI network visualized using Cytoscape (**Figure 4A**), module analysis conducted with the MCODE plugin identified two highest-scoring modules, each comprising seven genes (**Figure 4B**). The CytoHubba plugin was employed to rank nodes based on three centrality algorithms—DC, CC, and BC—each yielding a list of the top 10 genes (**Figure 4C**). The intersection between the 14 genes from the two top modules and the top 10 genes identified by each centrality algorithm resulted in the identification of six hub genes: SYK, CXCL8, TNF, NFKB1, PPARG and CASP8 (**Figure 4D**). GO enrichment analysis of the hub genes identified key biological processes relevant to RA, including inflammatory response, TNF−mediated signaling, activation of apoptotic cysteine−type endopeptidase activity, innate immune response, and death−inducing signaling complex assembly. These processes are closely linked to RA pathogenesis, where sustained TNF signaling and defective FLS apoptosis drive synovial hyperplasia and chronic inflammation. Cellular component analysis showed predominant enrichment in the cytoplasm, and molecular function analysis showed TNF receptor binding and protein−protein binding, supporting the role of these hub genes in signaling and transcriptional regulation (**Figure 4E**). KEGG pathway analysis revealed significant enrichment of pathways with established roles in RA, including NF−κB signaling, TNF signaling, apoptosis, Toll−like receptor signaling, NOD−like receptor signaling, and osteoclast differentiation. The convergence of TNF, NF−κB, and apoptosis pathways aligns with PIC’s known anti−inflammatory and pro−apoptotic activities, suggesting that PIC may suppress synovial inflammation and prevent bone erosion in RA (**Figure 4F**).

### 3.5 Molecular docking of the PIC and hub genes

**Figure 5:**
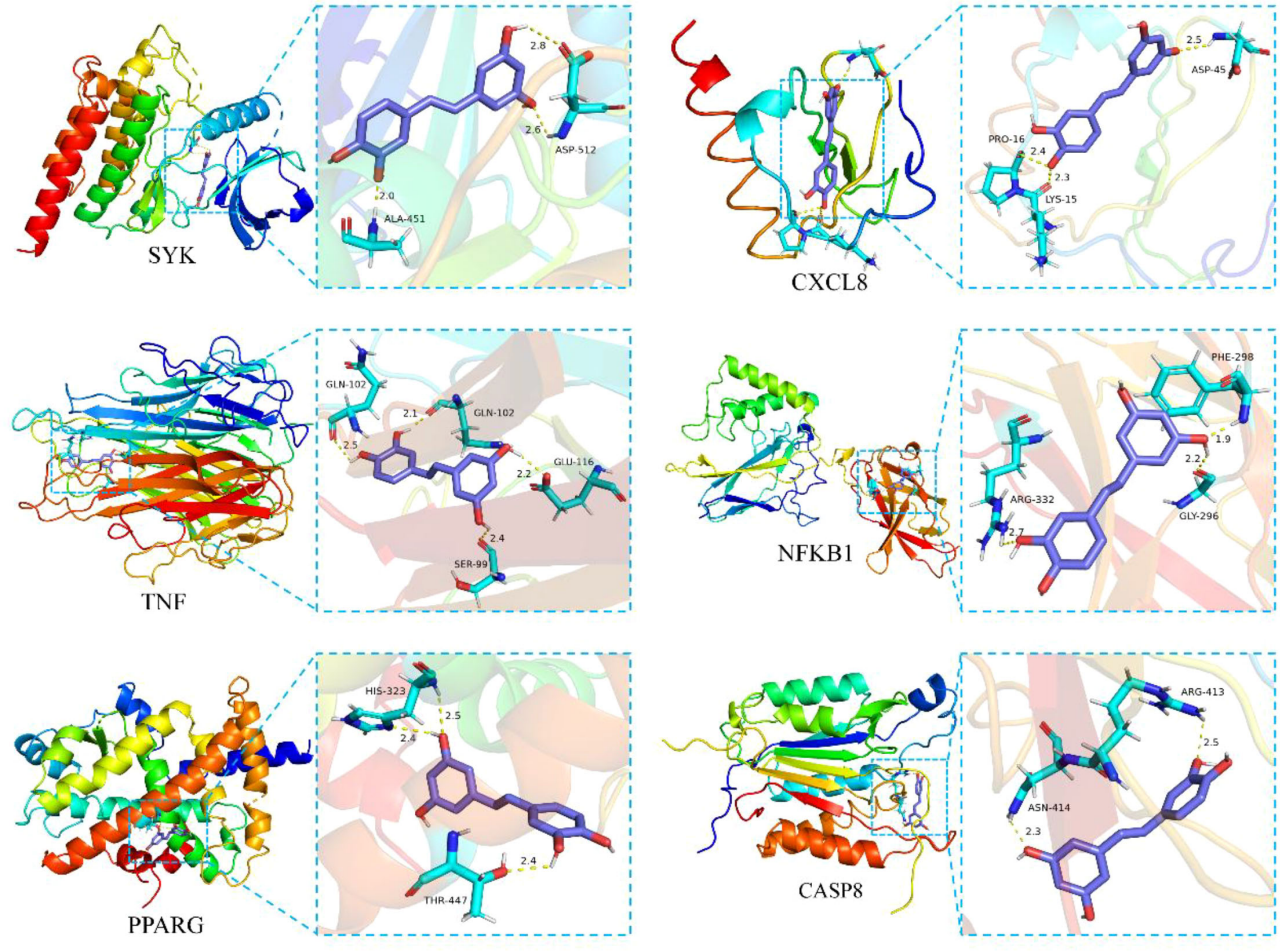
Molecular docking, PIC and the six hub genes, SYK, CXCL8, TNF, NFKB1, PPARG and CASP8.

Molecular docking was performed between PIC and the six identified hub genes. The dimensions of each connected grid box and key amino acid residues are shown in **Table 2**. The binding affinity of each molecular docking was less than 5.0 kcal/mol (**Table 2**). The results demonstrated binding affinity between PIC and each of these targets. These docking outcomes are consistent with the target predictions derived from network pharmacology, thereby validating the reliability of the bioinformatically inferred targets for PIC (**Figure 5**).

**Table 2:** Molecular docking of the PIC and hub genes.

Drug	Gene	Grid box dimension			Binding affinity	Key amino acid residues
		x	y	z		
PIC	SYK	23.8	20.8	18.9	-7.1	ALA451、ASP512
PIC	CXCL8	46.0	44.3	47.6	-5.6	PRO16、LYS15、ASP45
PIC	TNF	72.3	74.1	71.5	-7.7	GLN102、GLN102、GLU116、SER99
PIC	NFKB1	83.4	76.9	63.1	-6.9	ARG332、GLY296、PHE298
PIC	PPARG	54.2	61.2	74.0	-7.8	HIS323、THR447
PIC	CASP8	50.6	62.0	69.3	-6.7	ARG413、ASN414

### 3.6 Signature reversal analysis of PIC as a potential therapeutic agent for RA

**Figure 6:**
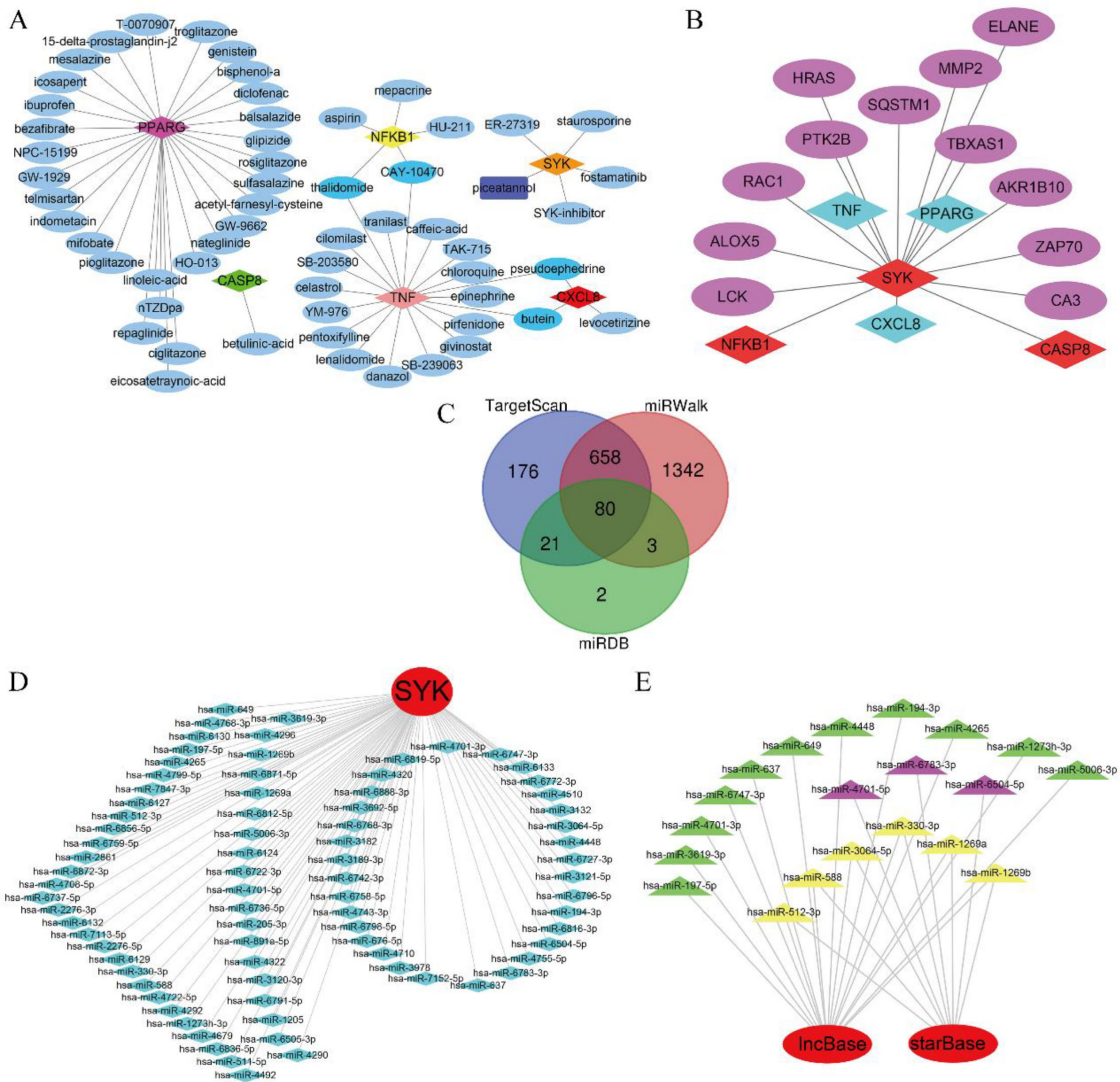
Further determination of PIC target genes and construction of the ceRNAs network. **(A)** Signature reversal analysis of the SYK and PIC; **(B)** Immune−related genes; **(C)** Identified miRNAs targeting SYK; **(D)** The intersection of the prediction results yielded 80 highly confident miRNAs; **(E)** The starBase and LncBase databases predict target lncRNAs.

Potential therapeutic agents targeting the six hub genes were systematically predicted. Specifically, CXCL8 was associated with 3 targeted drugs, TNF with 19, NFKB1 with 5, CASP8 with 1, and PPARG with 29. Among these, two overlapping drugs were identified between the sets for CXCL8 and TNF, and two additional overlaps were found between NFKB1 and TNF. Notably, SYK was predicted to have five targeted drugs, which included the investigational compound PIC (**Figure 6A**).

### 3.7 Molecular dynamics simulations

**Figure 7:**
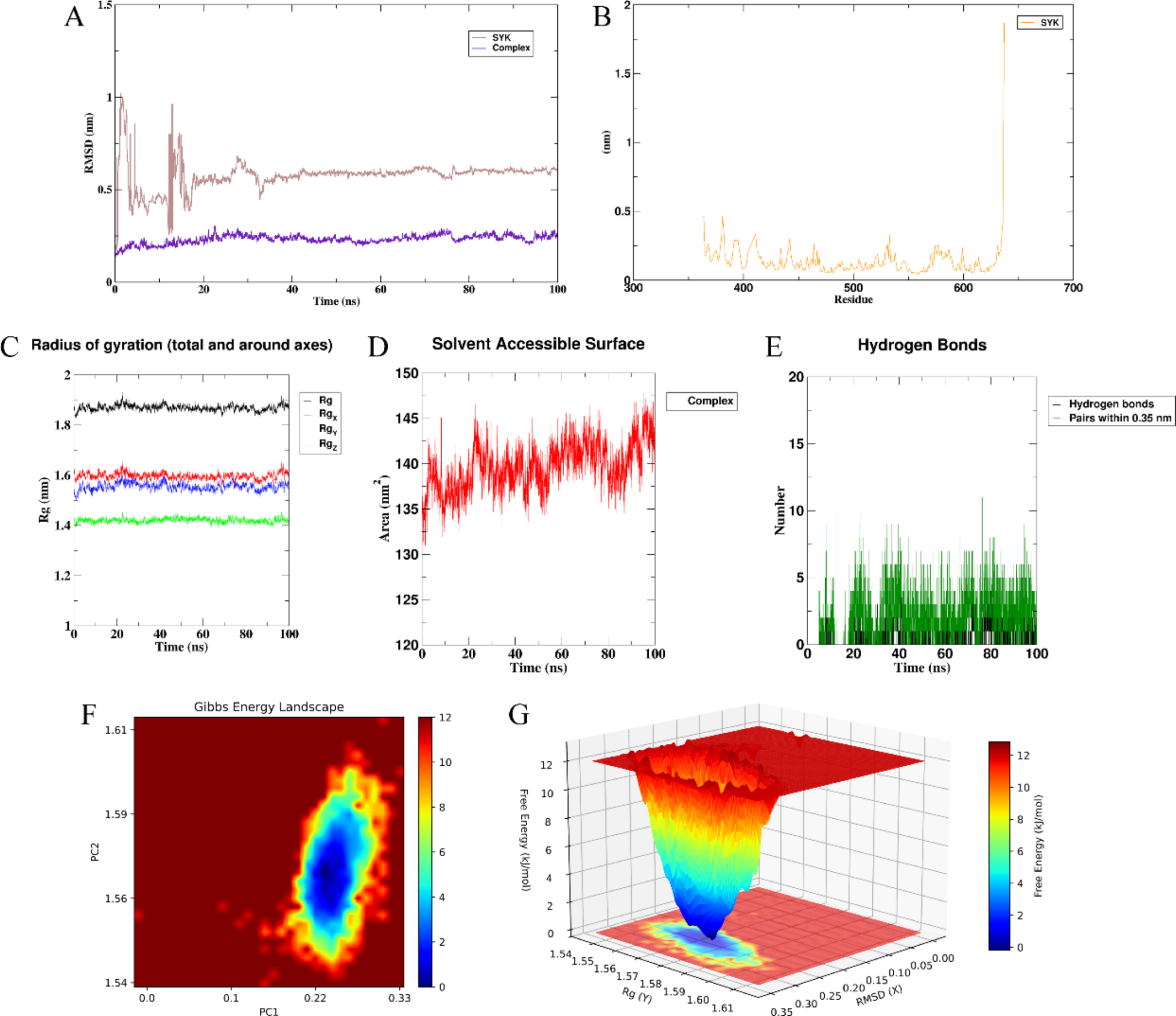
Molecular dynamics simulations of PIC and SYK. **(A)** Root-mean-square deviation; **(B)** Root-mean-square fluctuation; **(C)** Radius of gyration; **(D)** Olventaccessible surface area; **(E)** Number of protein–ligand hydrogen bonds; **(F-G)** Free energy landscape.

Molecular dynamics simulations were performed on PIC and SYK. RMSD analysis indicated that the RMSD of the complex stabilized after approximately 40 ns during the 100 ns simulation, suggesting high structural stability throughout the simulation (**Figure 7A**). RMSF values reflect the fluctuation of atoms or residues relative to the reference conformation during the simulation. Higher RMSF values are typically associated with greater structural flexibility and represent functional regions and binding sites of the protein (**Figure 7B**). Throughout the simulation, both complexes maintained stable Rg values without significant fluctuations, further demonstrating their structural integrity (**Figure 7C**). Moreover, the SASA values remained stable with minimal fluctuations, indicating that the protein tightly encapsulates the relevant molecule (**Figure 7D**). During the molecular dynamics simulation, hydrogen bonds were consistently formed between the complex and remained stable in number, indicating binding stability (**Figure 7E**). And illustrates the conformational free energy changes of the complex (**Figure 7F–G**). The molecular dynamics simulations demonstrate that the PIC–SYK complex maintains stable structural integrity, as evidenced by stable RMSD, Rg, SASA, and consistent hydrogen bonding throughout the 100 ns simulation.

### 3.8 Identification of immune−related genes and PIC target genes

From the 35 candidate target genes potentially involved in PIC treatment of RA, the 15 immune−related genes (immune score > 60) were selected based on the GeneCards database. The intersection between these immune-related genes and the previously identified hub genes yielded three overlapping genes—SYK, NFKB1, and CASP8—which were ultimately identified as potential core targets for PIC in the treatment of RA (**Figure 6B**).

### 3.9 Construction of the ceRNA network and screening of key regulatory axes

**Figure 8:**
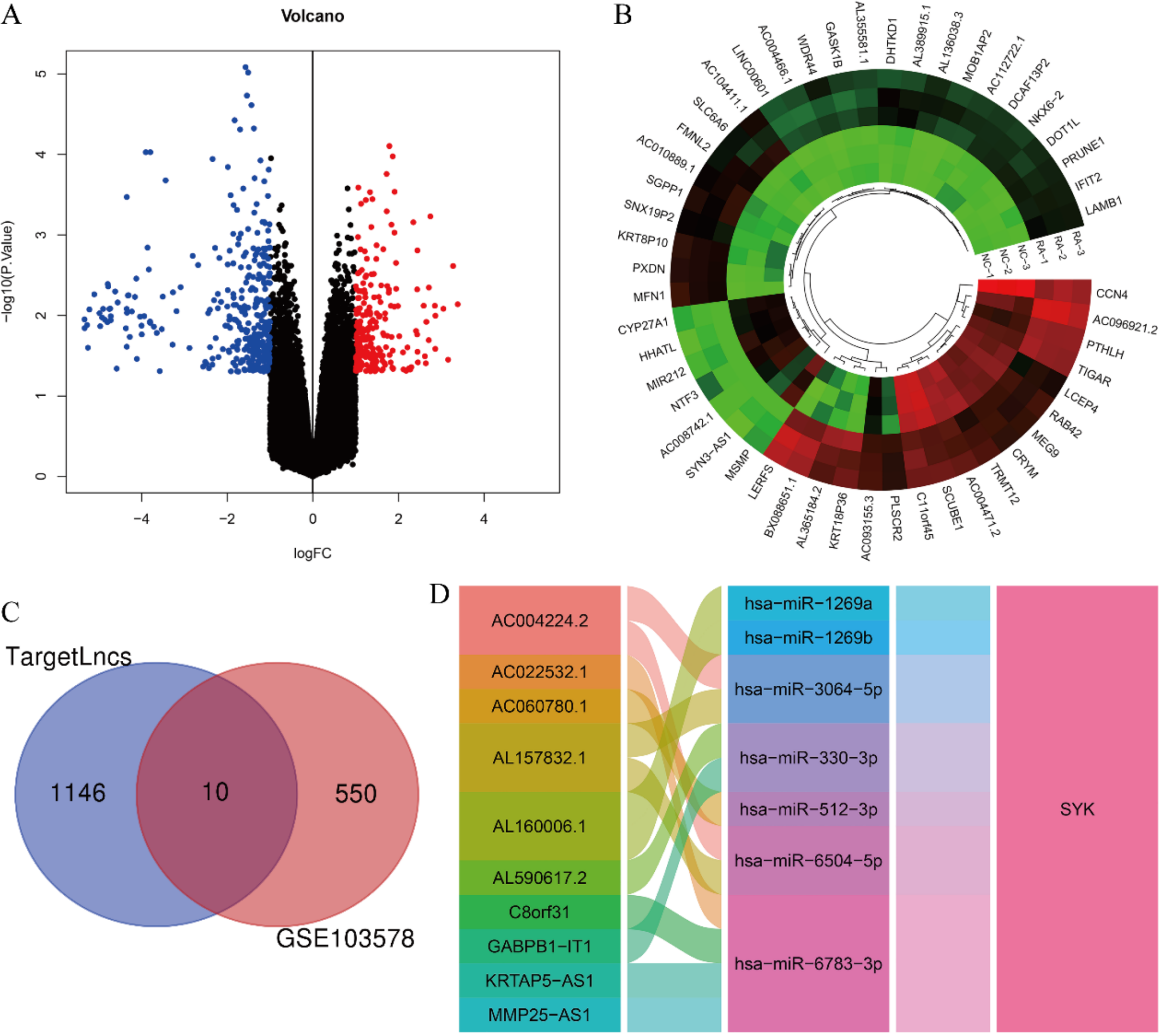
Key ceRNA regulatory axes. **(A)** Volcano plot of DEGs and DELncs in GSE103578; **(B)** Heatmap of DEGs and DELncs in GSE103578; **(C)** 10 candidate lncRNAs for PIC in RA treatment; **(D)** Key ceRNA regulatory axes.

Among the potential therapeutic targets for PIC in RA—SYK, NFKB1, and CASP8—SYK was selected for further investigation due to its capacity to be inversely predicted as a PIC target. A ceRNA network was subsequently constructed centered on SYK. Initial miRNA prediction identified 935, 2083, and 106 miRNAs targeting SYK in the TargetScan, miRWalk, and miRDB databases, respectively. The intersection of these predictions yielded 80 miRNAs (**Figures 6C, D**). Among these, 3 miRNAs were associated with target lncRNAs in starBase, 11 in LncBase, and 6 miRNAs overlapped between both databases (**Figure 6E**). A total of 1156 lncRNAs were predicted to be regulated by these 20 miRNAs. Meanwhile, analysis of the GSE103578 dataset identified 560 DEGs and DELncs, visualized via volcano plot (**Figure 8A**) and heatmap (**Figure 8B**). Intersection of the 1156 predicted lncRNAs with the 560 DEGs/DELncs yielded 10 candidate lncRNAs for PIC in RA treatment: AC022532.1, GABPB1-IT1, KRTAP5-AS1, AL590617.2, AC004224.2, C8orf31, MMP25-AS1, AL157832.1, AC060780.1, and AL160006.1 (**Figure 8C**). Based on these lncRNAs and their corresponding miRNAs and mRNAs, a ceRNA network was constructed, ultimately revealing 13 candidate ceRNA axes implicated in the mechanism of PIC for RA (**Figure 8D**).

### 3.10 Gene set enrichment analysis

**Figure 9:**
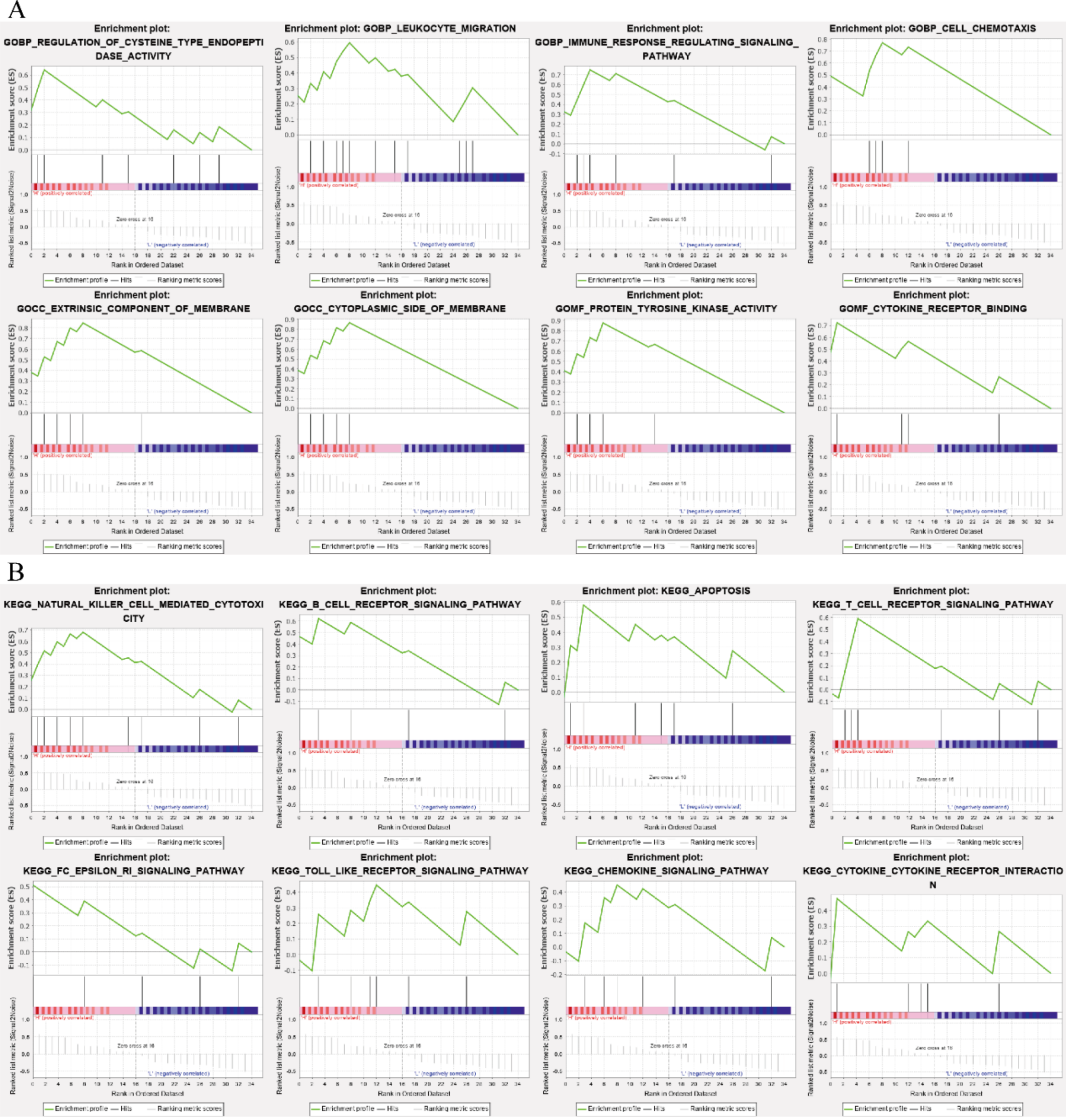
Gene set enrichment analysis of SYK; **(A)** GO enrichment analysis, include biological process, cellular component, and molecular function; **(B)** KEGG enrichment analysis.

GO enrichment analysis of SYK revealed its involvement in several biological processes critical to RA pathogenesis, including regulation of cysteine−type endopeptidase activity (implicated in apoptosis regulation), leukocyte migration, immune response−regulating signaling pathways, and cellular component—all of which contribute to synovial inflammation and immune cell infiltration. CC analysis showed enrichment at the cytoplasmic side of the membrane, consistent with SYK’s role as a proximal signaling kinase. Molecular function analysis showed protein tyrosine kinase activity and cytokine receptor binding, supporting SYK’s function in transducing pro−inflammatory signals (**Figure 9A**). KEGG pathway analysis demonstrated that SYK is prominently involved in the B cell receptor signaling pathway, T cell receptor signaling pathway, Fc epsilon RI signaling pathway, Toll−like receptor signaling pathway, and chemokine signaling pathway. These pathways are central to adaptive and innate immune responses in RA. Notably, the apoptosis pathway and natural killer cell−mediated cytotoxicity were also enriched, suggesting that SYK may regulate both immune cell survival and cytotoxic functions. Given that SYK is a known mediator of Fc receptor and B/T cell receptor signaling in RA synovium, its enrichment aligns with PIC’s potential to modulate immune−driven inflammation (**Figure 9B**).

### 3.11 PIC reduces the proliferation activity of MH7A cells

**Figure 10:**
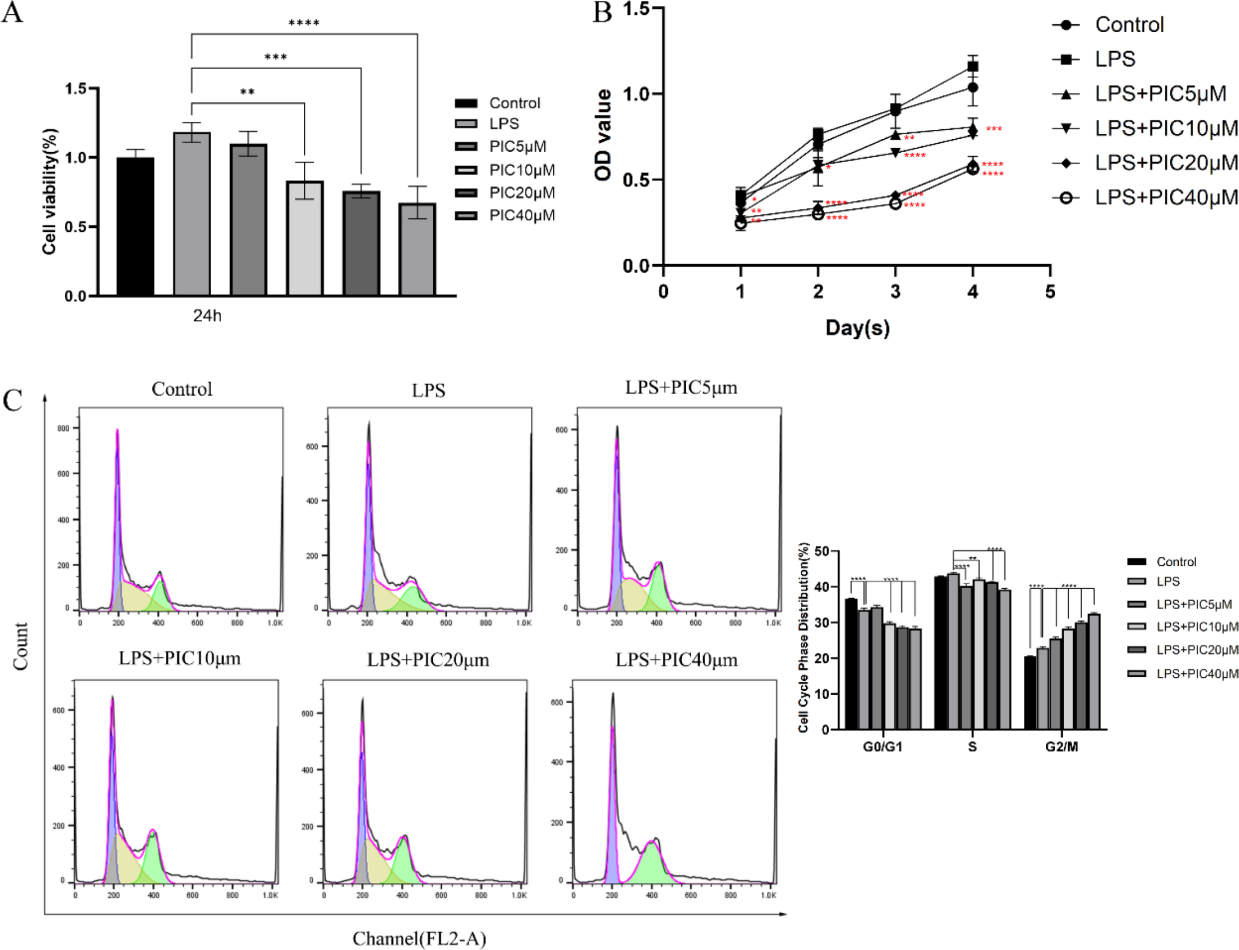
PIC reduces the viability of LPS-activated MH7A cells and induces cell cycle arrest at the G2/M phase. **(A)** 24-hour cell viability graph; **(B)** Cell survival line graph; **(C)** Representative flow cytometry curve graph and statistical graph. *p<0.05, **p<0.01, ***p<0.001, ****p<0.0001.

To evaluate the effect of PIC on the activity of LPS-activated MH7A cells, cells were treated with different concentrations of PIC (0, 5, 10, 20, and 40 μM) for 24 to 96 hours, then stimulated with 1 μg/mL LPS. Cell viability was determined by CCK-8 assay. After 24 hours, LPS alone did not significantly alter cell viability compared with the control group (98.25% ± 5.93%, p > 0.05). Co−treatment with PIC at 5μM did not significantly alter cell viability compared with the LPS alone (93.6% ± 4.67%, p > 0.05). In contrast, co−treatment with PIC at 10, 20, and 40 μM reduced cell viability to 72.78% ± 1.66%, 71.22% ± 3.05%, and 31.06% ± 4.54% of the LPS−alone group, respectively (p < 0.01), demonstrating a dose−dependent inhibitory effect. Similar trends were observed at 48, 72, and 96 hours, with increasing inhibition over time (**Figures 10A, B**).

### 3.12 PIC causes the cell cycle of MH7A cells to be arrested at the G2/M phase

We determined the cell cycle distribution of MH7A cells using a flow cytometer. In the control group, the proportions of cells in G0/G1 and G2/M phases were 36.61% ± 0.16% and 20.53% ± 0.05%, respectively. Treatment with LPS alone reduced the G0/G1 population to 33.46% ± 0.55% and increased the G2/M population to 22.86% ± 0.35% (p < 0.05). Co−treatment with PIC at 5, 10, 20, and 40 μM further decreased the G0/G1 phase to 32.26% ± 0.45%, 29.76% ± 0.48%, 28.67% ± 0.45%, and 28.33% ± 0.54%, respectively, while increasing the G2/M phase to 25.51% ± 0.40%, 28.22% ± 0.58%, 30.05% ± 0.29%, and 39.22% ± 0.27%, respectively (p < 0.0001). These results demonstrate that PIC induces a dose−dependent G2/M phase arrest in LPS−activated MH7A cells (**Figure 10C**).

### 3.13 The influence of PIC on apoptosis-related proteins

**Figure 11:**
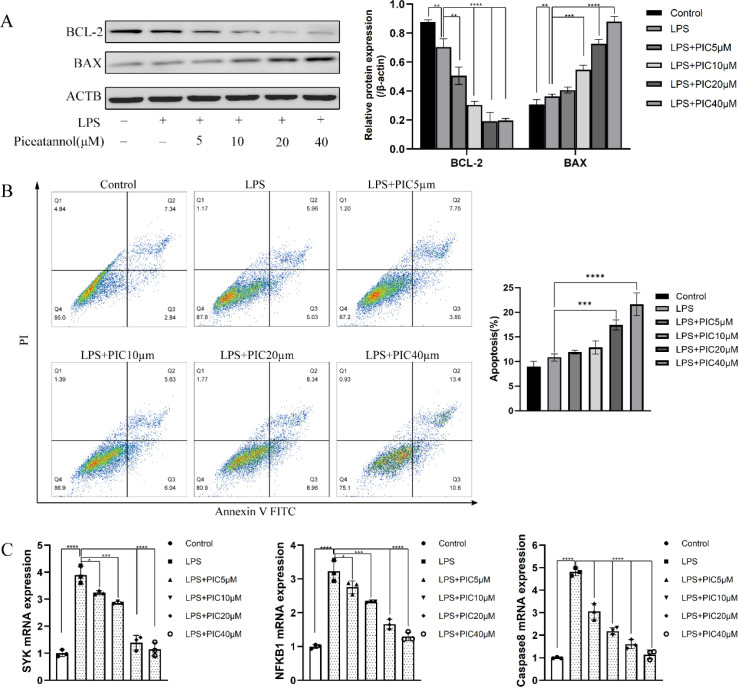
PIC promotes apoptosis of LPS-activated MH7A cells and verification of PIC’s effect on the expression levels of molecular targets. **(A)** Through Western blot analysis and statistical charts, study the impact of PIC on the expression of apoptosis-related proteins; **(B)** Representative flow cytometry curves and statistical charts; **(C)** Expression levels of molecular targets’ mRNA by PIC. *p<0.05, **p<0.01, ***p<0.001, ****p<0.0001.

To evaluate the effect of PIC on apoptosis-related proteins, the Western blot method was used to detect the influence of PIC on the expression of BAX and BCL-2. The Western blot results showed that, relative to the control group (BAX: [29.38% ± 1.82%], BCL-2: [87.78% ± 1.40%]), LPS treatment alone elevated the expression of BAX (to [38.94% ± 1.51%], p < 0.01) and reduced the expression of BCL-2 (to [70.35% ± 5.69%], p < 0.01). Furthermore, when compared with the LPS-only group, the combination of PIC and LPS further enhanced the expression of the pro-apoptotic protein BAX (to [87.91% ± 3.67%] at the highest PIC concentration) while diminishing the expression of the anti-apoptotic protein BCL-2 (to [19.62% ± 1.44%] at the highest PIC concentration), with both changes showing a clear dosage correlation (p < 0.0001) (**Figure 11A**). Then, flow cytometry was performed using Annexin V-FITC/PI, the effect of PIC on LPS-induced apoptosis of MH7A cells was detected. The apoptosis rate in the control group was 8.98% ± 1.05%. Following treatment with LPS alone for 24 hours, the apoptosis rate was (10.84% ± 0.72%, p > 0.05), showing no statistically significant difference compared with the control group. Likewise, combined treatment with PIC at 5 μM (11.90% ± 0.39%, p > 0.05) or 10 μM (12.85% ± 1.35%, p > 0.05) in the presence of LPS did not result in a significant change in apoptosis rate relative to LPS alone. In contrast, co-administration of PIC at 20 μM (17.40% ± 1.02%, p < 0.001) or 40 μM (21.61% ± 2.31%, p < 0.0001) with LPS significantly increased the apoptosis rate compared with LPS treatment alone (**Figure 11B**).

### 3.14 Validation of the expression levels of molecular targets by PIC

To verify the expression of the PIC-related molecular targets, the RT-qPCR method was used to detect the expression levels of the relevant molecules after PIC treatment. Relative to the control group (relative mRNA expression normalized to control = 1.00 ± 0.04), LPS treatment alone led to elevated mRNA expression levels of SYK (3.89 ± 0.33), CASP8 (4.81 ± 0.17), and NFKB1 (3.22 ± 0.31) in MH7A cells (p < 0.0001); in contrast, when compared with the LPS-only group, the combination of PIC and LPS lowered the mRNA expression levels of SYK (1.15 ± 0.26), CASP8 (1.14 ± 0.20), and NFKB1 (1.30 ± 0.13) (p < 0.0001), at the highest PIC concentration. Moreover, with increasing concentrations of PIC, the transcript levels of these three molecules progressively declined, exhibiting a clear concentration−response relationship (**Figure 11C**).

### 3.15 PIC reduces the severity of arthritis in AIA rats

**Figure 12:**
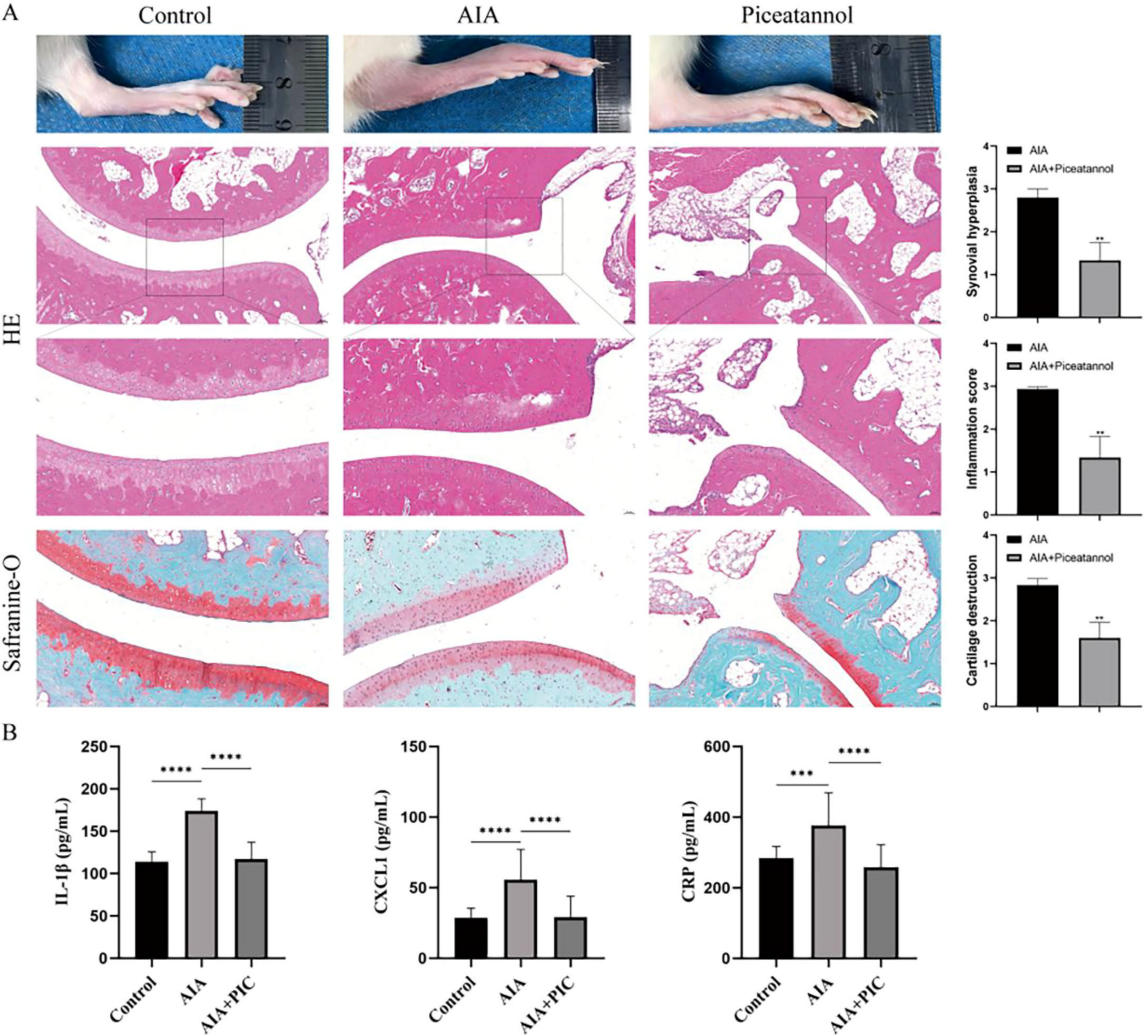
PIC improves the severity of arthritis in the RA rat model. **(A)** HE staining and Safranine-O staining images and statistical analysis results of the RA rat model (bar scale: 100 μm for 10×, 50 μm for 20×); **(B)** Serum IL-1β, CXCL1 and CRP levels of rats were measured using commercial ELISA kits. **p<0.01, ***p<0.001, ****p<0.0001.

To evaluate the potential anti-rheumatic effect of PIC, *in vivo* experiments were conducted in the AIA rat model. Compared with the normal control group, the joints of the AIA model group showed typical RA pathological features, including marked synovial hyperplasia, extensive inflammatory cell infiltration, and severe cartilage destruction. After treatment with PIC, these pathological changes were ameliorated, as evidenced by reduced synovial hyperplasia, decreased inflammatory cell infiltration, and less cartilage destruction. The statistical results of pathological scoring further quantitatively showed the above observations (**Figure 12A**). Additionally, serum levels of IL−1β, CXCL1, and CRP—key downstream effectors of the NF−κB signaling pathway—were measured using commercial ELISA kits. PIC treatment reduced the levels of these inflammatory cytokines in the arthritis rat serum (**Figure 12B**). Notably, these *in vivo* anti−inflammatory and joint−protective effects are consistent with our *in vitro* findings that PIC downregulates the mRNA expression of SYK and NFKB1 in MH7A cells, indicating that PIC likely exerts its therapeutic action in the AIA model through suppressing the SYK/NF−κB axis and subsequent reduction of pro−inflammatory mediators. Collectively, these *in vivo* results support that PIC is effective in treating RA, with a molecular mechanism involving inhibition of SYK/NF−κB signaling.

## 4 Discussion

Through bioinformatics analysis, we identified 35 candidate targets shared between PIC and RA−related DEGs, though this overlap depends on dataset quality and thresholds and does not imply direct functional relevance. PPI network analysis prioritized six hub genes (SYK, CXCL8, TNF, NFKB1, PPARG, CASP8), but such prioritization is algorithm−dependent. Molecular docking suggested in silico binding of PIC to all six hub genes, yet lacks biochemical validation. ConnectivityMap matched SYK specifically to PIC, reinforcing its therapeutic relevance, although this inference is limited by the database’s predictive nature and potential off−target effects. Intersecting the six hub genes with immune−specific gene sets yielded three core targets (SYK, NFKB1, CASP8), but the “immune−specific” definition is database−dependent and may introduce bias. *In vitro*, PIC dose−dependently downregulated SYK, NFKB1, and CASP8 mRNA in MH7A cells, correlating with reduced viability, G2/M arrest, increased BAX/BCL−2 ratio, and enhanced apoptosis. However, correlation does not prove direct regulation, as PIC may have off−target effects. In the AIA rat model, PIC alleviated synovial pathology and reduced serum IL−1β, CXCL1, and CRP (downstream effectors of NF−κB), but these findings do not distinguish whether effects are primarily mediated through SYK/NFKB1/CASP8. Collectively, the data suggest a hypothesis that PIC may inhibit the SYK/NF−κB axis to reduce inflammation and promote RA−FLS apoptosis, but this model remains speculative. The ceRNA network is entirely computational and requires functional validation. Future studies should use genetic manipulation and direct binding assays to establish causality. Until then, the conclusions should be viewed as hypothesis−generating.

A methodological limitation of this study is that no multiple testing correction was applied to the initial differential expression analysis. Given the high dimensionality of transcriptomic data, this threshold inevitably yields some false−positive DEGs. The intersection of PIC target genes (derived from databases) with these uncorrected DEGs may therefore include genes that are not genuinely associated with RA. However, several factors mitigate the potential impact of false−positive DEGs on the final conclusions. First, the uncorrected threshold (|logFC| ≥ 1, nominal P < 0.05) was used only as an initial filtering step to maintain sensitivity, rather than as a final criterion for target selection. Second, the candidate genes identified at this stage were subsequently subjected to multiple independent filters—including PPI network analysis, machine learning algorithms (LASSO and SVM−RFE), ConnectivityMap reverse matching, and immune−specific gene prioritization—which collectively reduce the likelihood that randomly arising false positives would survive through the entire pipeline. Third, and most importantly, the three hub genes (SYK, NFKB1, CASP8) were experimentally validated at the mRNA level in RA−MH7A cells, providing functional evidence beyond statistical inference. While the absence of FDR correction remains a limitation, the convergence of computational predictions with experimental results supports the biological relevance of these targets. We recommend that future studies confirm these findings using larger cohorts with appropriate multiple testing correction.

PIC is known as a selective inhibitor of SYK, and previous studies have demonstrated its therapeutic effects in experimental arthritis and its anti−inflammatory actions in RA−FLS (25, 26). However, these prior investigations have mostly been limited to experimental validation of single signaling pathways. The present study offers a complementary perspective by integrating multiple computational and experimental layers rather than discovering an entirely novel function of PIC. Specifically, we employed a systematic “funnel−shaped” multi−level research paradigm that combines network pharmacology, PPI network analysis, molecular docking, ConnectivityMap reverse matching, and ceRNA network construction – progressing from global target screening to focused validation. Using this approach, we observed that PIC simultaneously downregulates three immune−related hub genes – SYK, NFKB1, and CASP8 – in RA−FLS, and we noted that this concurrent downregulation correlates with G2/M arrest, increased BAX/BCL−2 ratio, enhanced apoptosis, and reduced levels of NF−κB−driven inflammatory cytokines (IL−1β, CXCL1, CRP) *in vivo*. These correlations are consistent with the possibility of a SYK/NF−κB−related mechanism, but they do not prove causality. Additionally, we constructed a SYK−centered ceRNA regulatory network (13 candidate axes) for PIC in RA, which provides a hypothetical framework for post−transcriptional regulation that may generate testable hypotheses. These findings require functional validation (e.g., genetic manipulation, luciferase assays). The integrated strategy and the observed correlations represent a modest methodological extension relative to previous single−pathway validation studies, rather than on new biological insights. We emphasize that all mechanistic interpretations remain strictly hypothesis−generating and should not be taken as established conclusions.

Among the three potential therapeutic targets for PIC in RA—SYK, NFKB1, and CASP8—SYK was prioritized for further ceRNA network analysis based on a combination of criteria. First, ConnectivityMap analysis revealed that PIC could be inversely predicted as a potential modulator of SYK, suggesting a regulatory relationship between PIC and SYK expression. Second, among the three candidates, SYK serves as a proximal tyrosine kinase in Fc receptor and B/T cell receptor signaling pathways, both of which are central to RA pathogenesis. Third, while NFKB1 and CASP8 are also critical for inflammation and apoptosis respectively, SYK has been more extensively implicated as a druggable target in autoimmune arthritis, and its inhibition is known to reduce synovial inflammation and bone erosion. Given these rationales, we selected SYK for in−depth mechanistic exploration via ceRNA network construction. It should be noted, however, that NFKB1 and CASP8 remain highly relevant to PIC’s anti−RA effects and warrant future investigation.

In the present study, we observed that PIC dose−dependently reduced the viability of MH7A cells, induced G2/M phase arrest, and promoted apoptosis, as evidenced by increased BAX/BCL−2 ratio and Annexin V−positive cells. Concurrently, PIC downregulated the mRNA expression of SYK, NFKB1, and CASP8—three hub genes identified through network pharmacology and molecular docking. Notably, SYK is a proximal tyrosine kinase that activates NF−κB signaling (27), while NFKB1 encodes the p50 subunit of NF−κB, a master transcription factor driving the expression of pro−inflammatory and anti−apoptotic genes (28). CASP8 is an initiator caspase in the extrinsic apoptosis pathway (29). Based on these correlations, one possible interpretation is that PIC downregulates SYK and NFKB1, which in turn may suppress NF−κB activity, leading to reduced production of inflammatory mediators (e.g., IL−1β, CXCL1) and decreased expression of anti−apoptotic proteins such as BCL−2, thereby shifting the balance toward apoptosis. The reduction in CASP8 expression could appear counterintuitive for apoptosis induction; however, CASP8 can also exert non−apoptotic functions (30), and its downregulation might reflect a feedback mechanism or a cell−type−specific response. Alternatively, PIC may primarily act through the mitochondrial (BAX/BCL−2−driven) pathway, with CASP8 playing a secondary role. In the AIA rat model, PIC treatment alleviated synovial hyperplasia, inflammatory infiltration, cartilage destruction, and serum levels of IL−1β, CXCL1, and CRP—all downstream effectors of NF−κB. Collectively, these observations are consistent with a speculative model in which PIC targets the SYK/NF−κB axis and modulates CASP8 expression, potentially contributing to cell cycle arrest, apoptosis of RA−FLS, and attenuation of joint inflammation and erosion. This integrated framework links computational predictions, cellular phenotypes, and *in vivo* outcomes, but it remains a hypothesis−generating model rather than a proven mechanism. Experimental validation through genetic knockdown, selective inhibitors, and protein−level analyses is required to establish causality.

SYK is a well−established non−receptor tyrosine kinase broadly expressed in hematopoietic cells, mediating immune receptor signaling and regulating proliferation, differentiation, and phagocytosis (27, 31). Previous studies have demonstrated that SYK deficiency protects against autoantibody−induced arthritis in mice (32), and intra−articular delivery of SYK−knockdown cells suppresses arthritis progression (33). Elevated SYK phosphorylation in peripheral blood B cells of ACPA−positive RA patients (34), together with the efficacy of the SYK inhibitor R788 in reducing joint damage in biologic−refractory RA patients (35, 36), suggests that SYK may be a clinically relevant target. PIC has been reported to act as a selective SYK inhibitor in hematopoietic and non−hematopoietic contexts (37). While these prior findings establish SYK as a promising anti−RA target, the present study adds several correlative observations. Using network pharmacology and PPI analysis, we identified SYK as a hub gene among PIC’s predicted targets. Molecular docking quantified the predicted binding affinity between PIC and SYK. We observed that PIC dose−dependently downregulates SYK mRNA expression in MH7A cells. Additionally, we noted associations between SYK downregulation and functional outcomes (G2/M arrest, increased BAX/BCL−2 ratio, enhanced apoptosis) as well as *in vivo* efficacy (reduced synovial pathology and NF−κB−driven inflammatory mediators). These integrated observations are consistent with a hypothesis in which PIC treatment leads to SYK/NFKB1 suppression, reduced inflammation, increased apoptosis, and amelioration of arthritis. However, it must be acknowledged that our study does not provide direct evidence (e.g., SYK knockdown/overexpression or kinase activity assays) to prove that the observed anti−RA effects are causally dependent on SYK inhibition. Therefore, while our data are compatible with SYK as a potential mediator of PIC’s action, definitive causality remains to be established in future studies.

NFKB1 is a critical endogenous modulator of the NF−κB pathway, regulating immune and inflammatory responses (38). In RA, the NFKB1 promoter polymorphism rs28362491 is associated with disease severity and cardiovascular comorbidity (39, 40), and NFKB1 expression levels correlate with clinical disease activity (41). In the present study, we identified NFKB1 as a predicted target of PIC through network pharmacology, and we observed that PIC dose−dependently downregulates NFKB1 mRNA expression in MH7A cells. Furthermore, PIC treatment reduced serum levels of IL−1β, CXCL1, and CRP in the AIA rat model—all established downstream effectors of NF−κB signaling. These observations are compatible with the possibility that PIC may influence NF−κB−driven inflammatory responses, potentially through effects on NFKB1 expression. Our study only measured NFKB1 mRNA levels; we did not assess protein expression, nuclear translocation of NF−κB subunits, or DNA−binding activity. We did not use a selective NF−κB inhibitor or NFKB1 knockdown/overexpression to directly test whether the observed cellular and *in vivo* effects are causally dependent on NFKB1 downregulation. The reduction in downstream inflammatory cytokines could also result from PIC’s effects on other pathways. Therefore, while our data provide correlative evidence linking PIC to NFKB1 modulation, the inference that PIC ameliorates RA via NFKB1 remains preliminary and hypothesis−generating, requiring functional validation in future studies.

CASP8 encodes an apical caspase that mediates apoptosis and suppresses RIPK3/MLKL−dependent necroptosis (29). In RA, the CASP8 variant G regulates invasive behavior in RA−FLS, and selective CASP8 inhibition reduces cell invasion without the adverse effects of pan−caspase inhibition (42). Additionally, Angelica and Pain Decoction induces RA−FLS apoptosis via the Fas/CASP8 pathway (43). In the present study, we identified CASP8 as a predicted target of PIC and observed that PIC dose−dependently downregulates CASP8 mRNA expression in MH7A cells, which correlates with increased apoptosis (elevated BAX/BCL−2 ratio and Annexin V positivity). However, this correlation does not demonstrate causality. Several caveats prevent any causal conclusion. First, CASP8 downregulation would typically be expected to reduce apoptosis, yet we observed the opposite. This discrepancy suggests that the observed apoptosis is unlikely to be mediated by CASP8 activation; instead, PIC may primarily act through the mitochondrial (BAX/BCL−2−driven) pathway, with CASP8 changes possibly being an epiphenomenon. Second, we did not measure CASP8 enzymatic activity, cleaved caspase−8, or downstream caspase−3/7 activation. Third, no genetic manipulation (CASP8 knockdown/overexpression) or selective caspase−8 inhibitor was used to establish causality. Therefore, the proposed involvement of CASP8 in PIC’s anti−RA mechanism remains speculative. Future studies employing CASP8−specific siRNA, CRISPR knockout, or selective inhibitors combined with functional readouts are required to determine whether CASP8 plays any necessary role in PIC−induced apoptosis in RA−FLS.

Through ceRNA network construction, we identified 13 candidate regulatory axes centered on SYK. These axes involve several miRNAs (e.g., hsa−miR−330−3p, hsa−miR−512−3p, hsa−miR−1269a/b) and lncRNAs (e.g., GABPB1−IT1, KRTAP5−AS1, MMP25−AS1, AC060780.1). However, the vast majority of existing evidence for these molecules comes from cancer studies. For instance, GABPB1−IT1 is downregulated in non−small cell lung cancer and associated with poor prognosis (44, 45). KRTAP5−AS1 serves as a prognostic marker in papillary thyroid carcinoma (46). MMP25−AS1 has been linked to immune infiltration in renal cell carcinoma (47). AC060780.1 is implicated in breast cancer progression among HIV patients (48). Similarly, hsa−miR−330−3p is differentially expressed in esophageal squamous cell carcinoma (49), hsa−miR−512−3p acts as a tumor suppressor in breast cancer (50), and hsa−miR−1269a promotes non−small cell lung cancer (51). To date, no direct functional studies have linked these ceRNA components to rheumatoid arthritis pathogenesis or to PIC’s anti−arthritic effects. Notably, the functional roles of several predicted molecules (e.g., AC022532.1, AC004224.2, C8orf31, AL157832.1, hsa−miR−6504−5p) remain unreported. Therefore, this ceRNA network is entirely computational and lacks any experimental validation in the context of RA. It should not be interpreted as evidence of biological function or mechanism. Instead, it may serve as a source of candidate hypotheses for future experimental testing (e.g., luciferase assays, siRNA−mediated knockdown). Any functional relevance of these axes remains unknown at this stage.

However, this study also has certain limitations. Firstly, the differential expression analysis and enrichment analysis did not perform multiple test correction, which may increase the risk of false positives. This is consistent with the research methods in previous literature, and similar previous studies also did not perform multiple monitoring correction (18, 19). Secondly, the molecular docking results suggesting binding between PIC and the hub genes are purely computational and lack experimental validation (e.g., surface plasmon resonance or competitive binding assays with known inhibitors). Therefore, the claim of “binding affinity” remains speculative at this stage. Thirdly, the ceRNA network analysis involved multiple prediction layers without experimental validation. Therefore, the biological relevance of these ceRNA axes remains uncertain, and false-positive associations cannot be excluded. Future studies should prioritize experimental verification of the most promising axes before drawing mechanistic conclusions. Fourthly, the use of a single cell line (MH7A) reduces the general applicability. Fifthly, core target validation was limited to mRNA levels; we did not assess protein expression or perform functional perturbation experiments (e.g., siRNA−mediated knockdown of SYK, NFKB1, or CASP8, or selective pharmacological inhibitors). Thus, the proposed mechanism remains largely correlative rather than causal, as we cannot determine whether the observed anti−proliferative and pro−apoptotic effects of PIC are directly dependent on these targets. Future studies should include protein−level validation, genetic manipulation, and pathway−specific inhibition to establish causality and to confirm the SYK/NF−κB axis in primary RA−FLS from multiple donors. Sixthly, the silico predictions (such as SwissTargetPrediction) are prone to false positives, and the intersection approach (PIC targets and DEGs) may oversimplify complex biology by assuming that differentially expressed genes are direct therapeutic targets. Thus, the overlapping genes should be viewed as hypothesis−generating candidates rather than validated targets.

## 5 Conclusion

In summary, this study combined network pharmacology with experimental validation to investigate PIC in RA. We identified 35 candidate targets, prioritized six hub genes (SYK, CXCL8, TNF, NFKB1, PPARG, CASP8), and showed in silico binding. *In vitro*, PIC reduced MH7A cell viability, induced G2/M arrest and apoptosis, and downregulated SYK, NFKB1, and CASP8 mRNA. In AIA rats, PIC alleviated synovial pathology and reduced serum IL−1β, CXCL1, and CRP. These findings suggest PIC may exert anti−RA effects potentially via the SYK/NF−κB axis and apoptosis pathways. However, limitations include lack of direct biochemical validation of docking, unproven causality (genetic perturbation needed), purely computational ceRNA network, and use of a single cell line and animal model without pharmacokinetic or safety assessment. Thus, claims that PIC could serve as an RA therapeutic are premature. This study is hypothesis−generating, providing a theoretical basis and testable hypotheses for future research, which should prioritize target validation, causality testing, and more rigorous preclinical evaluation.

## Data Availability

The datasets presented in this study can be found in online repositories. The names of the repository/repositories and accession number(s) can be found in the article/supplementary material.
